# Investigating the association between blood metabolites and telomere length: A mendelian randomization study

**DOI:** 10.1371/journal.pone.0298172

**Published:** 2024-03-08

**Authors:** Chen Gao

**Affiliations:** Head and Neck Surgeons, Clinical Oncology School of Fujian Medical University, Fujian Cancer Hospital, Fujian, China; Brigham and Women’s Hospital and Harvard Medical School, UNITED STATES

## Abstract

**Background:**

Telomere length refers to the protective cap at the end of chromosomes, and it plays a crucial role in many diseases. The objective of this study is to explore the relationship between blood metabolites and telomere length, aiming to identify novel biological factors that influence telomere length.

**Methods:**

In this study, we extracted genome-wide association study (GWAS) data for blood metabolites from a sample of 7824 Europeans. Additionally, GWAS data for telomere length were obtained from the Open GWAS database (GWAS ID: ieu-b-4879). The primary analysis of this study utilized the random inverse variance weighted (IVW) method. Complementary analyses were also conducted using the MR-Egger and weighted median approaches. Sensitivity analyses were performed to assess the robustness of the findings. These included the Cochran Q test, MR-Egger intercept test, MR-PRESSO, and leave-one-out analysis. To investigate the possibility of reverse causation, reverse MR analysis was conducted. Additionally, multivariable MR was utilized to evaluate the direct effect of metabolites on telomere length.

**Results:**

The results suggested a potential association between 15-methylpalmitate, taurocholate, levulinate, and X-12712 and telomere length. MVMR analysis further showed that 15-methylpalmitate, taurocholate, and levulinate can directly influence telomere length, regardless of other metabolites.

**Conclusions:**

This study suggests that 15-methylpalmitate, taurocholate, and levulinate are likely factors correlated with telomere length. These findings will contribute to the development of strategies for protecting telomeres, preventing related diseases, and establishing a new biological foundation for achieving healthy aging.

## Introduction

Telomere length, the protective cap at the end of chromosomes, is closely associated with the occurrence, development, and prognosis of many diseases [1–3].Recent studies have found that telomere length is associated with various diseases [4–6], including age-related conditions [7,8], metabolic disorders [9], tumors [10,11], neurological diseases[12], immune system disorders, respiratory diseases [13], psychosis [14], cardiovascular diseases [15], and digestive system diseases [16]. Deepening our understanding of the relationship between telomere length and these diseases can help reveal the mechanisms of disease development and provide new biological foundations for early diagnosis, risk assessment, and treatment. Telomere length may also emerge as an important biomarker in clinical practice. Telomere length is influenced by a variety of factors, such as gender [17], age [18], telomerase activity [19], oxidative stress [20], lifestyle factors [21,22] (such as smoking, alcohol consumption, and poor dietary habits), genetic factors [23], environmental factors [24], and disease states [25]. Understanding these influencing factors can help us better protect telomeres, prevent related diseases, and provide a new biological basis for healthy aging.

Blood metabolites, which are metabolic products present in the blood, have a significant impact on telomere length. Telomere length, in turn, affects cell aging, immune function, and disease development. These metabolites encompass lipids [26], amino acids, carbohydrates [27], nucleotides, and more. For instance, some metabolites like oxidized low-density lipoprotein (oxLDL) [28] and phosphatidylcholine [29] may be associated with shorter telomeres, while anti-inflammatory lipids such as ω-3 polyunsaturated fatty acids (PUFA) [30] and phosphatidylserine [31] may have a protective effect on telomeres, lengthening their length. Additionally, certain amino acid metabolites like branched-chain amino acids (BCAAs) [32], aromatic amino acids (AAs) [33], and glutathione (GSH) [34] are also linked to telomere length. Hyperglycemia has been found to be associated with telomere shortening, and improving glucose metabolism can help safeguard telomeres [35,36]. Furthermore, nucleotides, including thymidine nucleotide, can impact telomere length [37]. By deepening our understanding of the relationship between blood metabolites and telomere length, we can gain insights into the role of metabolites in cell aging, disease development, and progression. Ultimately, this knowledge can pave the way for new strategies in the prevention and treatment of related diseases.

Mendelian randomization (MR) is a method used to investigate evidence of causal relationships [38]. It leverages the random distribution of genetic variations at the time of conception to minimize residual confounding and reverse causality. By utilizing genetic variations as instrumental variables (IVs) [39] that are strongly associated with risk factors, MR can effectively assess the causal effects of metabolites on telomere length. MR avoids the confounding factors present in traditional epidemiological studies by using genetic variations as tools, thereby revealing robust causal relationships. Moreover, MR benefits from large sample sizes, enabling more precise and reliable identification of metabolites associated with telomere length.

This study aimed to explore the association and causal relationship between blood metabolites and telomere length using a Mendelian randomization approach. We utilized valid genetic variants associated with 486 blood metabolites, extracted from published genome-wide association study (GWAS) data. Our research design and analysis aimed to provide valuable insights into the complex relationship between blood metabolites and telomere length. By gaining a deeper understanding of the potential impact of blood metabolites on telomere length, we may uncover new pathways through which blood metabolites can influence health.

## Materials and methods

This study employed summary statistics extracted from a genome-wide association study. The initial investigations had acquired ethical endorsement and informed consent from the participants, as confirmed by the institutional review boards. Since this analysis did not encompass any novel data collection or necessitate supplementary ethical clearance, obtaining further ethical approval or informed consent specifically for this study was unnecessary.

### Study design

**Fig 1:**
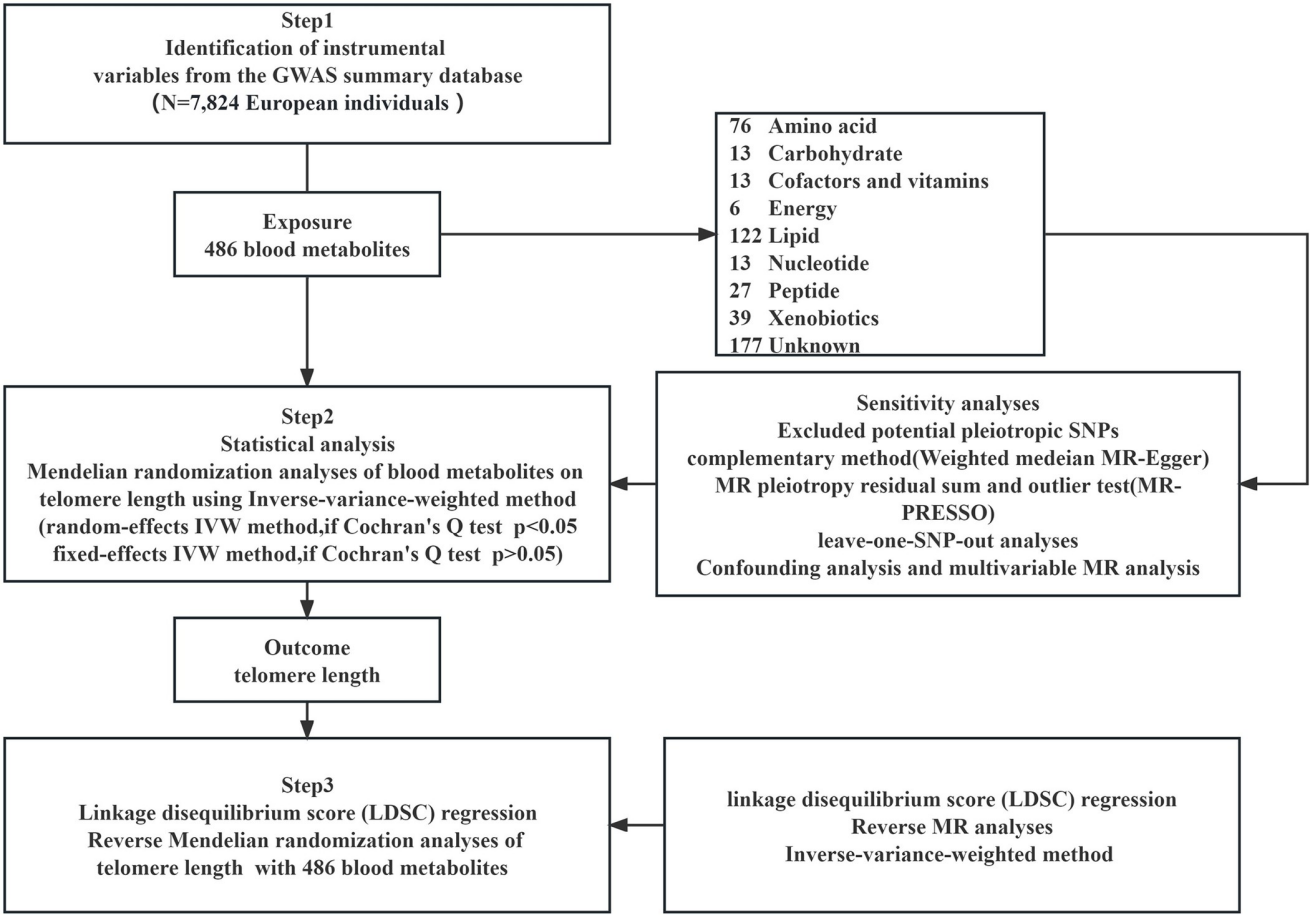
A graphical representation of the study.

Fig 1 illustrates the complete blueprint of the current investigation. The construction of this study took inspiration from a previous MR study [40,41].

### GWAS data for 486 blood metabolites and telomere length

The genetic data of blood metabolites in this study were acquired from the metabolomics GWAS server (https://metabolomics.helmholtz-muenchen.de/gwas/), which provides a comprehensive compilation of genetic loci linked to blood metabolites. This dataset is considered the most extensive available information concerning genetic variation associated with blood metabolites [42]. It furnishes detailed data on genetic variants and their correlation with specific blood metabolites. The chemical composition of the 486 metabolites is listed in S1 Table in S1 File. It is crucial to mention that some of these metabolites have an unidentified chemical structure. Conversely, others have been validated and assigned into eight broad categories [43]: amino acid, carbohydrate, cofactors and vitamins, energy, lipid, nucleotide, peptide, and xenobiotic metabolism. GWAS information pertaining to telomere length was procured from the Open GWAS database (https://gwas.mrcieu.ac.uk/, GWAS ID: ieu-b-4879).

### IVs selection

To ensure the quality and consistency of instrumental variables (IVs) in accordance with the core assumptions of Mendelian randomization (MR), we implemented various quality control measures. Initially, we selected independent SNPs strongly associated with blood metabolites, maintaining a p-value threshold of <5 × 10–8. However, we were unable to find SNPs that met both the blood metabolite and telomere length criteria, so we adjusted the IV selection threshold to 1 × 10–5. We then used a clustering method based on European ancestry individuals from the 1000 Genomes Project (1000G EUR) [44] to exclude SNPs with strong linkage disequilibrium (LD), setting an r2 < 0.01 and a window size of 500 kb. This method has been widely used in previous studies [45,46]. Among the SNP pairs that exceeded the threshold, we only retained those with low p-values to reduce bias from weak instruments. To further minimize bias, we calculated the F statistics for each SNP and excluded those with F < 10 as poor genetic variants. Ambiguous SNPs with allelic incongruence and median allele frequency were also removed to ensure effect allele consistency. In the reverse MR analysis, we used a stricter threshold (p < 5 × 10–8) [47] to select IVs associated with telomere length. If certain SNPs were not available in the outcome summary data, we refrained from searching for proxy SNPs. This decision was based on the understanding that a small proportion of missing SNPs has a limited impact on the results [48]. In order to fulfill the criterion that independent variables (IVs) must have no association with the outcome, we eliminated IVs that showed a significant association with the outcome (p < 1×10–5). Finally, to ensure effect allele concordance, we excluded SNPs with ambiguous alleles and intermediate allele frequencies. These carefully selected SNPs will be used as the final genetic IVs for subsequent MR analysis.

### Statistical analysis and sensitivity analysis

In this study, we investigated the relationship between blood metabolites and telomere length using three different evaluation methods: the inverse variance weighting (IVW) method [49], the weighted median (WM) method [50], and the MR-Egger method [51]. The IVW method assumes no horizontal pleiotropy for all SNPs and provides the most accurate assessment of causal effects. The MR-Egger and WM methods complement the IVW method by allowing the detection of horizontal pleiotropy and the inclusion of invalid SNPs. Together, these methods offer a more reliable and comprehensive analysis of the relationship between blood metabolites and telomere length. We calculated odds ratios (OR) and their corresponding 95% confidence intervals (CI) to determine the association between 486 blood metabolites and telomere length. To address the issue of multiple comparisons, we applied the Bonferroni correction method and set the significance threshold at p < 0.000103 (Bonferroni correction with 486 tests) to account for the large number of metabolites analyzed. If a p-value fell between the significance threshold and 0.05, it indicated suggestive evidence for a potential causal association [52].

In Mendelian randomization (MR) studies, conducting sensitivity analysis is crucial for examining potential bias that can significantly impact MR estimates, such as horizontal pleiotropy and heterogeneity. To ensure reliable estimates, our study employed several sensitivity analysis tests. Firstly, the Cochran Q test was employed for identifying heterogeneity within the results [53]. Next, the MR-Egger intercept was computed to examine directional pleiotropy and potential bias arising from invalid instrumental variables [51]. Additionally, we employed the MR-Pleiotropy Residuals and Outliers (MR-PRESSO) method to identify and adjust for heterogeneous SNPs, thereby reducing potential confounding effects that could impact MR estimates [54]. Furthermore, we conducted a leave-one-out (LOO) analysis to assess the robustness of our findings by evaluating the individual impact of each SNP on the overall results. Through these sensitivity analyses, our aim was to identify and address possible biases, thereby strengthening the reliability of our findings.

In summary, to ensure the reliability of our findings, we followed strict criteria during the Mendelian randomization analysis process. Firstly, the primary analysis should yield a significant p-value (IVW derived p < 0.05). Secondly, we expected to observe consistent direction and magnitude of effect across all three MR methods (IVW, MR-Egger, and WM methods). Thirdly, there should be no evidence of heterogeneity or horizontal pleiotropy in the MR results. Lastly, we aimed to avoid heavy influence of a single SNP on the MR estimates. By adhering to these rigorous criteria, our objective was to identify blood metabolites with strong evidence supporting their potential causal association with telomere length. All MR analyses were conducted using R version 4.3.1 (https://www.r-project.org/) with the "Mendelian Randomization," "Two Sample MR," and "MR-PRESSO" packages.

### Evaluation of genetic correlation and directionality

Genetic correlations between exposures and outcomes can introduce bias in Mendelian randomization (MR) estimates [55,56]. Even if we exclude single nucleotide polymorphisms (SNPs) associated with telomere length when selecting instrumental variables (IVs), there remains a possibility that other SNPs not linked to telomere length may indirectly influence the results of the MR analysis. To address this concern, we employed linkage disequilibrium score (LDSC) regression [57] to investigate whether there exists a genetic correlation between the blood metabolites examined in our study and telomere length. This approach helps ensure that the observed causal effects are not attributable to co-heritability between the exposure and outcome. Additionally, we conducted reverse MR analysis and the Steiger test [58] to assess potential bias arising from reverse causality. Through the combined use of LDSC analysis, the Steiger test, and reverse MR analysis, our aim was to enhance the reliability of our findings while minimizing potential bias stemming from genetic correlation or reverse causation.

### Confounding analysis and multivariable MR analysis

It is important to acknowledge that our study may still be affected by residual confounding due to genetic variation that is not well understood or measured. To further evaluate the relationship between each SNP in our instrumental variable (IV) and known risk factors for telomere length, such as age-related conditions, tumors, neurological diseases, immune system disorders, psychosis, cardiovascular diseases, and digestive system diseases, we conducted an analysis using the Phenoscanner database (http://www.phenoscanner.medschl.cam.ac.uk/). Each SNP was individually examined to determine if there was a significant association with these confounders (p < 1 ×10–5). If any SNPs were found to be significantly associated with the confounders, they were excluded, and the MR analysis was repeated. This additional step was implemented to enhance the credibility of our study by minimizing the potential impact of confounding effects. In practical analyses, some genetic variants may exhibit pleiotropy [59], meaning they are associated with multiple risk factors or traits. To address this issue, we utilized multivariable Mendelian randomization (MVMR) to assess potential interactions between the identified metabolites. By utilizing MVMR analysis [60], our aim was to account for potential confounding issues arising from pleiotropy and interactions. These methods significantly strengthened the causal inferences in our analysis of the relationship between blood metabolites and telomere length risk, while effectively addressing the complexities associated with pleiotropy and interactions.

## Results

### Main results of the association between 486 blood metabolites and telomere length

After rigorous control for the quality of instrumental variables (IVs), 486 metabolites were analyzed in the Mendelian randomization (MR) study. All single nucleotide polymorphisms (SNPs) associated with these metabolites exhibited F statistics greater than 10, indicating strong instrumental variable power. The detailed data of the IVs can be found in S2 Table in S1 File. The MR results for the associations between all 486 metabolite traits and telomere length are presented in S3 Table in S1 File. Initially, the inverse variance weighted (IVW) method identified 27 metabolites that potentially have causal effects on telomere length. Among these, 21 metabolites have known chemical identities and belong to categories such as lipid, xenobiotics, amino acid, cofactors and vitamins, peptide, carbohydrate, and energy. Additionally, 6 metabolites have unknown chemical identities. Further details can be found in S2 Table in S1 File. Following complementary and sensitivity analyses, four metabolites that met rigorous screening criteria were selected for further analysis (Table 1). These include taurocholate (OR 0.97, 95% confidence interval [CI]: 0.96–0.99, p = 0.0018), 15-methylpalmitate (OR 0.89, 95% confidence interval [CI]: 0.81–0.98, p = 0.0189), levulinate (OR 1.06, 95% CI: 1.01–1.12, p = 0.0254), and X-12712 (OR 0.99, 95% CI: 0.98–1.00, p = 0.0133).

**Table 1:** Statistical and sensitivity analyses for causality from blood metabolites on telomere length in Mendelian randomization (MR) analysis.

	MR analysis			Heterogeneity	Pleiotropy		PubChem	HMDB
Metabolite	method	p	OR(95%CI)	p	Intercept	p		
Lipid								
15-methylpalmitate (isobar with 2-methylpalmitate)	IVW	0.0189	0.89 (0.81–0.98)	0.0556	-0.0001	0.96	17903417	HMDB0061709
	WM	0.3151	0.94 (0.84–1.06)					
	ME	0.3510	0.89 (0.71–1.12)					
taurocholate	IVW	0.0018	0.97 (0.96–0.99)	0.5098	-0.0006	0.54	6675	HMDB0000036
	WM	0.0219	0.97 (0.95–1.00)					
	ME	0.1176	0.98 (0.95–1.00)					
Amino acid								
levulinate (4-oxovalerate)	IVW	0.0254	1.06 (1.01–1.12)	0.3383	0.0002	0.97	5177120	HMDB0061709
	WM	0.2438	1.06 (0.96–1.17)					
	ME	0.3136	1.06 (0.95–1.19)					
Unknown								
X-12712	IVW	0.0133	0.99 (0.98–1.00)	0.1728	-0.0007	0.63		
	WM	0.3172	0.99 (0.98–1.01)					
	ME	0.3776	0.99 (0.98–1.01)					

**Fig 2:**
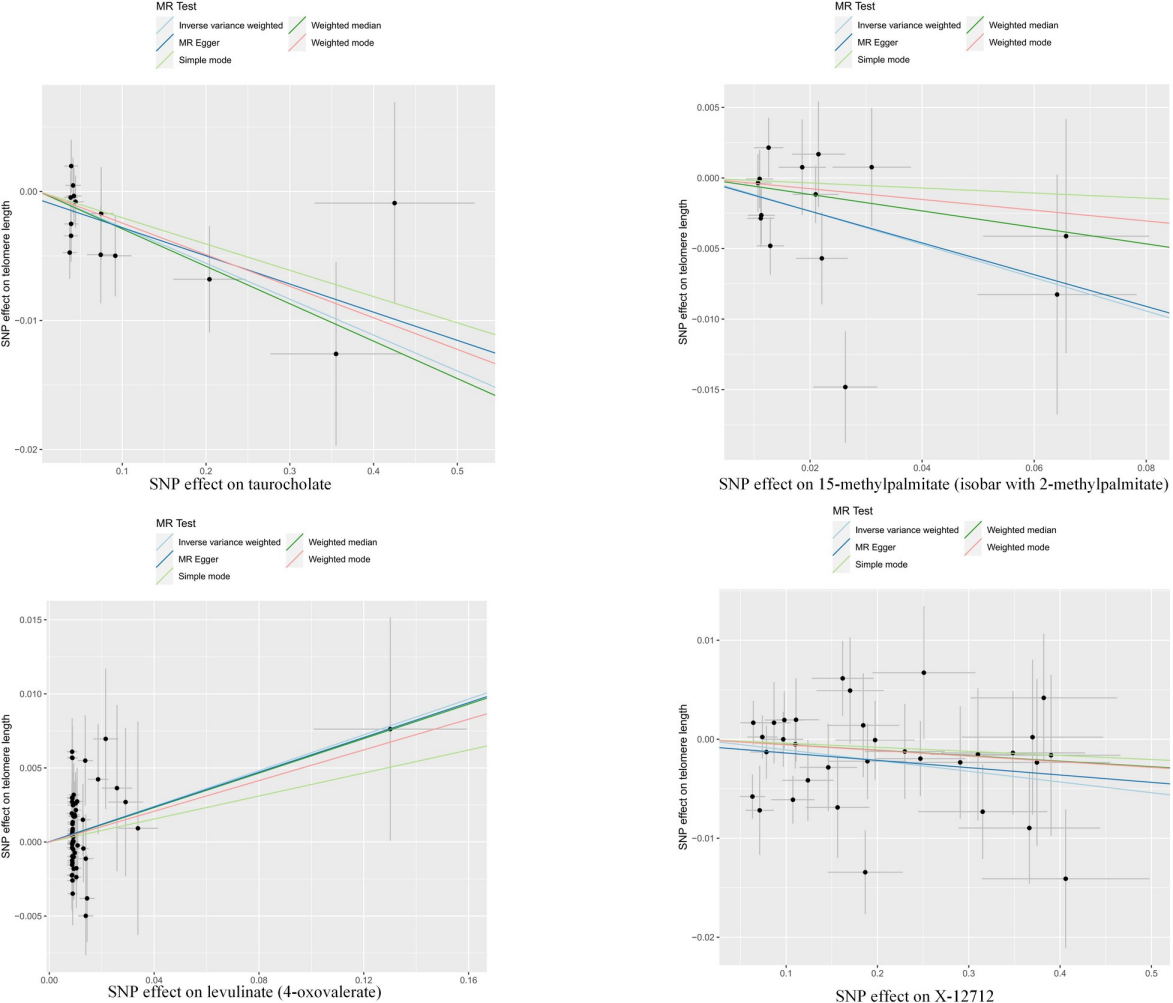
Scatterplot of significant associations (derived using inverse variance weighted method; p-value < 0.05) and consistent estimates in MR analyses.

**Fig 3:**
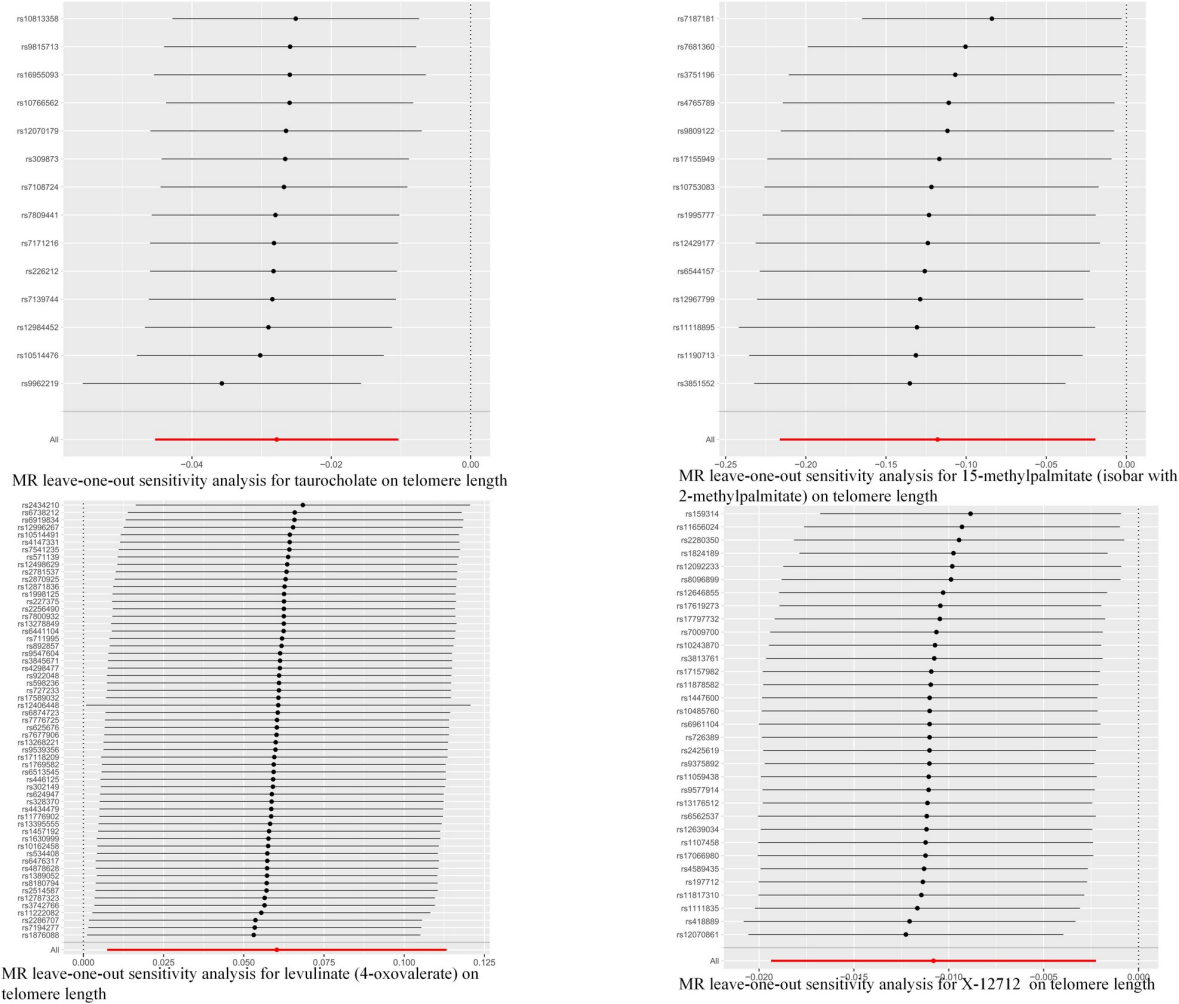
Leave-one-out plot to visualize causal effect of blood metabolites on telomere length when leaving one SNP out.

In summary, the estimates obtained from the inverse variance weighted (IVW) method were found to be statistically significant (p < 0.05). Additionally, the estimates derived from IVW, MR-Egger, and weighted median (WM) methods were consistent in both direction and magnitude, as illustrated in Fig 2. The MR-PRESSO results presented in S4 Table in S1 File, which involved the removal of outliers, also did not support the presence of heterogeneous single nucleotide polymorphisms (SNPs). The absence of heterogeneity and pleiotropy was convincingly supported by the Cochran Q test (p > 0.05) and MR-Egger intercept test (p > 0.05), as demonstrated in Table 1. Moreover, the LOO analysis results illustrated that there was no bias introduced in the estimation of Mendelian randomization (MR) due to a single SNP, as presented in Fig 3. Based on these findings, the four blood metabolites examined are considered promising candidates for further analysis of their potential causal effects on telomere length.

### Evaluation of genetic correlation and directionality

In this study, LDSC-based estimates were used to assess the genetic correlation between telomere length and three specific metabolites: 15-methylpalmitate, taurocholate, and levulinate. The results showed a minimal genetic correlation between telomere length and these metabolites (15-methylpalmitate: Rg = -0.027, Se = 0.063, p = 0.666; taurocholate: Rg = -0.161, Se = 0.126, p = 0.202; levulinate: Rg = 0.022, Se = 0.036, p = 0.536). In addition, the LDSC analysis estimated the SNP-heritability of these metabolites. SNP-heritability refers to the proportion of variation in the levels of metabolites that can be linked to single nucleotide polymorphisms (SNPs) across the entire genome. The estimated SNP-heritability values were 0.276 for 15-methylpalmitate, 0.099 for taurocholate, and 0.858 for levulinate, as shown in S5 Table in S1 File. To investigate the potential reverse associations between these blood metabolite traits and telomere length, reverse Mendelian randomization (MR) analyses were conducted. The inverse variance weighted (IVW) method was used, and the results from S6 Table in S1 File indicate that there were no statistically significant associations between telomere length and any of these blood metabolite traits. Additionally, the Steiger test was performed to assess causality between genetically proxied metabolites and telomere length, and the results from S3 Table in S1 File show that there was no violation of causality due to reverse causal effects. These findings suggest that there is no evidence of reverse causality between genetically proxied metabolites and telomere length.

### Confounding analysis and MVMR

In order to ensure the independence of potential confounding factors, an exhaustive examination was carried out using the Phenoscanner website (http://www.phenoscanner.medschl.cam.ac.uk/) to verify the instrumental variables (IVs) employed in this study. Each individual SNP associated with these metabolites was carefully scrutinized to determine if they also exhibited associations with common influences factors impacting telomere length, such as age, telomerase activity, oxidative stress, lifestyle factors (e.g., smoking, alcohol consumption, and poor diet), genetic factors, environmental factors, and disease states. A total of 11 SNPs were identified with associations to traits unrelated to these blood metabolites (S7 Table in S1 File). These pleiotropic SNPs were subsequently excluded, and the associations between these four blood metabolites and telomere length were recalculated. Remarkably, the results remained consistent in the IVW method, demonstrating stability (S8 Table in S1 File). Following the adjustment for metabolite interactions, multivariable Mendelian randomization (MVMR) analysis was performed utilizing the IVW methods. The outcomes unveiled that 15-methylpalmitate, taurocholate, and levulinate, as genetically predicted, possess the ability to directly influence telomere length independent of other metabolites (S9 Table in S1 File).

## Discussion

This study was the first to investigate the potential relationship between blood metabolites and telomere length. Using a rigorous Mendelian randomization design, we discovered that certain blood metabolites might be causally associated with telomere length. Specifically, we found a positive correlation between levulinate and telomere length, while taurocholate and 15-methylpalmitate were negatively correlated with telomere length. These findings contribute to a better understanding of telomere protection and the prevention of related diseases.

The present study suggested a positive correlation between levulinate and telomere length. As a metabolite, the specific mechanism by which levulinate regulated telomere length remained to be further elucidated. Based on relevant research, levulinate might influence telomere length through several potential pathways. Firstly, it might participate in energy metabolism processes, such as the citric acid cycle (TCA cycle) [61] and oxidative phosphorylation [62], generating the cellular energy (ATP) required for telomere maintenance. Secondly, levulinate might be involved in biosynthetic pathways, such as nucleotide and amino acid synthesis, which were closely related to telomere biogenesis and maintenance [37,63]. Lastly, it might exhibit antioxidant properties [64], capable of reducing the production of intracellular oxygen free-radical [65], thereby protecting telomeres from oxidative damage and influencing telomere length maintenance [66,67]. To fully understand this association, future research should further explore these potential mechanisms and delve into the effects of levulinate on telomere length in various disease backgrounds and related risk factors.

In this study, we observed a negative correlation between taurocholate and telomere length. Taurocholate, a blood metabolite, previously lacked clear evidence for its specific impact on telomere length. However, given the significant role of bile acids in cell signaling and lipid metabolism [68], we hypothesized that taurocholate might influence telomere length by regulating cell growth, differentiation, apoptosis [69–71], and altering intracellular fatty acid and cholesterol levels [28,30,72]. Additionally, taurocholate was primarily synthesized and metabolized in the liver, and liver dysfunction could lead to abnormal bile acid metabolism, subsequently affecting telomere length [73]. However, these proposed mechanisms required further scientific research for validation. To fully understand the specific effects of these metabolites on telomere length, further detailed research might be needed under experimental conditions. This might include delving into the mechanism of action of taurocholate at the cellular and molecular levels and examining the impact of taurocholate on telomere length in different disease backgrounds and its associated risk factors.

This study discovered that 15-methylpalmitate, as a saturated fatty acid, was negatively associated with telomere length. It could influence telomere length through various mechanisms [74], such as participating in cellular energy metabolism, affecting mitochondrial function and fatty acid metabolism, regulating antioxidant enzyme expression and activity, altering the intracellular redox status [75], triggering inflammatory responses, influencing the generation and release of inflammatory factors, and affecting immune cell activity [76,77]. Additionally, it could regulate cell signaling pathways such as Wnt, Notch, PI3K/Akt [78], and affect DNA methylation and histone modification epigenetic processes [79], thereby influencing telomere length [80,81]. However, these proposed mechanisms required further scientific research for confirmation.

This study presented several notable advantages. Firstly, it was the most exhaustive and methodical investigation conducted until then, specifically focusing on exploring the cause-effect association between blood metabolites and telomere length. The analysis examined 486 blood metabolites, ensuring a comprehensive exploration of the subject. Secondly, Secondly, stringent MR analytical methods were utilized to tackle potential limitations encountered in previous research, such as reverse causality and confounding variables. Various methods were used to generate reliable estimates and eliminate any factors that may undermine MR analysis assumptions. The consistency observed in the direction of the three MR estimates and the robustness exhibited in sensitivity analyses further enhanced the credibility of the findings. Lastly, to further improve the reliability of our findings, we adopted multivariate Mendelian randomization (MVMR) and evaluated the heritability of IV, as well as the genetic correlation between metabolites and telomere length using LDSC. This supplementary verification process added strength to the conclusions derived from the MR analysis.

This study presented several limitations that needed to be acknowledged from an academic perspective. Firstly, given the limited number of SNPs that reached genome-wide significance, we decided to employ a relaxed P threshold instead of relying on genome-wide significance, which was a common practice in similar situations. Consequently, it is vital to highlight that this relaxed threshold may have elevated the possibility of false positive results. However, it is worth noting that all the selected SNPs exhibited F-statistic values exceeding 10, indicating the reliability of our instrumental variables (IV). Additionally, the consistent causal direction inferred through Steiger tests, reverse MR analysis, and the resulting outcomes further reaffirmed the credibility of our relaxed threshold setting. Secondly, although MR studies typically advocated for large GWAS sample sizes, we utilized relatively small metabolite GWAS sample sizes in our study, which could potentially influence the robustness of our MR study results. A third limitation lay in the fact that our MR analysis solely relied on GWAS data from individuals of European ancestry. As a result, it was imperative to exercise caution when generalizing these findings to other racial groups, and further investigation into this matter was warranted. Fourthly, detecting the metabolites with positive results in this study might have posed a challenge in actual testing. However, in this Mendelian random analysis, we did not observe any statistical significance when comparing the easily detectable upstream and downstream metabolites that were examined with regards to telomere length. It was important to emphasize that other upstream and downstream metabolites were not included in the 486 blood metabolite dataset due to the limited number of metabolites available in the GWAS data. In future studies, we intended to delve deeper into investigating the correlation between these additional upstream and downstream metabolites and telomere length. Lastly, a significant Bonferroni-corrected p-value threshold was used in this study, but none of the results reached this threshold. The study considered findings from other relevant studies and recognized that results with p-values between 0.05 and the Bonferroni correction threshold provided suggestive evidence of a potential association. Therefore, further research was needed to confirm the relationship between metabolites and telomere length. Consequently, despite the valuable insights offered by MR analysis concerning the etiology, it was crucial to stress the requirement for rigorous validation of our findings through randomized controlled trials and fundamental investigations before their clinical applicability could be established.

## Conclusions

In conclusion, our study using the Mendelian randomization method has shown that three genetically-proxied blood metabolites have an impact on telomere length. This significant discovery contributes to our knowledge of the biological processes involved in telomere regulation, offering valuable insights for the development of strategies aimed at protecting telomeres, preventing diseases associated with telomere dysfunction, and promoting healthy aging.

## Supporting information

S1 File S1-S10 Tables are included in file. (XLS)
